# Hepcidin induces intestinal calcium uptake while suppressing iron uptake in Caco-2 cells

**DOI:** 10.1371/journal.pone.0258433

**Published:** 2021-10-13

**Authors:** Supathra Phoaubon, Kornkamon Lertsuwan, Jarinthorn Teerapornpuntakit, Narattaphol Charoenphandhu

**Affiliations:** 1 Doctor of Philosophy Program in Biochemistry (International Program), Faculty of Science, Mahidol University, Bangkok, Thailand; 2 Department of Biochemistry, Faculty of Science, Mahidol University, Bangkok, Thailand; 3 Center of Calcium and Bone Research (COCAB), Faculty of Science, Mahidol University, Bangkok, Thailand; 4 Department of Physiology, Faculty of Medical Science, Naresuan University, Phitsanulok, Thailand; 5 Department of Physiology, Faculty of Science, Mahidol University, Bangkok, Thailand; 6 Institute of Molecular Biosciences, Mahidol University, Nakhon Pathom, Thailand; 7 The Academy of Science, The Royal Society of Thailand, Dusit, Bangkok, Thailand; University of Massachusetts Lowell, UNITED STATES

## Abstract

Abnormal calcium absorption and iron overload from iron hyperabsorption can contribute to osteoporosis as found in several diseases, including hemochromatosis and thalassemia. Previous studies in thalassemic mice showed the positive effects of the iron uptake suppressor, hepcidin, on calcium transport. However, whether this effect could be replicated in other conditions is not known. Therefore, this study aimed to investigate the effects of hepcidin on iron and calcium uptake ability under physiological, iron uptake stimulation and calcium uptake suppression. To investigate the potential mechanism, effects of hepcidin on the expression of iron and calcium transporter and transport-associated protein in Caco-2 cells were also determined. Our results showed that intestinal cell iron uptake was significantly increased by ascorbic acid together with ferric ammonium citrate (FAC), but this phenomenon was suppressed by hepcidin. Interestingly, hepcidin significantly increased calcium uptake under physiological condition but not under iron uptake stimulation. While hepcidin significantly suppressed the expression of iron transporter, it had no effect on calcium transporter expression. This indicated that hepcidin-induced intestinal cell calcium uptake did not occur through the stimulation of calcium transporter expression. On the other hand, 1,25(OH)_2_D_3_ effectively induced intestinal cell calcium uptake, but it did not affect intestinal cell iron uptake or iron transporter expression. The 1,25(OH)_2_D_3_-induced intestinal cell calcium uptake was abolished by 12 mM CaCl_2_; however, hepcidin could not rescue intestinal cell calcium uptake suppression by CaCl_2_. Taken together, our results showed that hepcidin could effectively and concurrently induce intestinal cell calcium uptake while reducing intestinal cell iron uptake under physiological and iron uptake stimulation conditions, suggesting its therapeutic potential for inactive calcium absorption, particularly in thalassemic patients or patients who did not adequately respond to 1,25(OH)_2_D_3_.

## Introduction

Low bone mass, osteopenia and osteoporosis were shown in several diseases with iron overload, such as hereditary hemochromatosis and β-thalassemia [1–7]. While iron overload and intestinal iron hyperabsorption were reported, the ineffective calcium absorption was also found in these patients and animal models [1, 8–11]. Correspondingly, previous study from our group also showed reduced bone mass and bone formation in thalassemic mice. Furthermore, intestinal iron hyperabsorption but ineffective calcium absorption was also found in these animal models [9]. Interestingly, a decreased level of serum 1,25(OH)_2_D_3_, which is a potent stimulator of intestinal calcium absorption, was noted in thalassemia patients [6, 12–14]. However, standard 1,25(OH)_2_D_3_ supplementation could not effectively rescue intestinal calcium malabsorption in thalassemic animals [15]. Because intestinal iron hyperabsorption was evident in animal models and patients with iron overload-associated osteoporosis, the alteration in intestinal iron transport regulation in these cases was also elucidated. Under physiological conditions, intestinal iron transport was negatively controlled could be suppressed by a small peptide called hepcidin. Hepcidin is a peptide hormone produced by hepatocytes and has been shown to inhibit iron transport by reducing iron recycling in macrophages and inhibiting iron absorption in enterocytes [16–21]. Hepcidin regulates systemic iron level by manipulating iron transporter and iron transport-associated protein expression, particularly divalent metal transporter 1 (DMT1) and ferroportin (FPN) [22–24]. Results from our group also showed that hepcidin could effectively relieve intestinal iron hyperabsorption in thalassemic mice. To our surprise, we also found that ineffective calcium absorption could significantly be improved by the iron transport suppressor, hepcidin, in thalassemic mice [15]. Several complications affecting intestinal iron and calcium absorption, including iron overload, abnormal liver function and impair vitamin D metabolism, were shown in thalassemia [1, 25, 26]. However, whether hepcidin-induced intestinal calcium uptake could be seen in other conditions beside thalassemia is largely unknown, and whether 1,25(OH)_2_D_3_ also affects intestinal iron transport system is still unclear. In addition, whether hepcidin could also rescue calcium absorption in other conditions remains to be investigated. Accordingly, this study aimed (*i*) to investigate the effects of hepcidin on intestinal cell iron and calcium uptake capacity under physiological and under iron uptake stimulation conditions, (*ii*) to demonstrate the effects of hepcidin on the expression of iron and calcium transporters in intestinal cells, (*iii*) to examine the effects of 1,25(OH)_2_D_3_ on intestinal cell iron uptake ability, (*iv*) to investigate the effects of 1,25(OH)_2_D_3_ on the expression of iron transporters and (*v*) to investigate the effects of hepcidin on intestinal cell calcium uptake under calcium uptake suppression by CaCl_2_. This study has thus provided information regarding the effectiveness and the conditions that hepcidin could be used to improve intestinal calcium absorption. This will be the crucial step for the application of hepcidin to alleviate both iron hyperabsorption and ineffective calcium absorption simultaneously.

## Material and method

### Cell culture

Human intestinal epithelium-like Caco-2 cells, which are the widely use intestinal epithelial cell models for intestinal ion transport studies, was used in this study [27–30]. Caco-2 cells (no.HTB-37, American Type Culture Collection, ATCC, Manassas, VA, USA) were obtained from Center of Calcium and Bone research (COCAB), Faculty of Science, Mahidol University. Caco-2 cells were cultured in Dulbecco’s modified Eagle’s medium (DMEM; Gibco, Waltham, MA, USA) supplemented with 15% (v/v) fetal bovine serum (FBS; Gibco), 1% non-essential amino acid (NEAA; Gibco), 1% l-glutamine (Gibco) and 1% Penicillin Streptomycin (Gibco). They were maintained at 37°C with 5% CO_2_ and sub-cultured when cells reached 95% confluence with 1× trypsin (Gibco). The medium was changed twice a week.

### Chemical and reagents

Human hepcidin (PLP-4392-S; Peptide institute, Osaka, Japan), 1,25(OH)_2_D_3_ (Cayman Chemical, Ann Arbor, MI, USA), L-Ascorbic acid 2-phosphate sesquimagnesium salt hydrate (L-Ascorbic; A8960; Sigma-Aldrich, Saint Louis, MO, USA) and calcium chloride (CaCl_2_; Sigma-Aldrich) were used. Ferric ammonium citrate (FAC; Sigma-Aldrich) was utilized as a donor of ferric iron.

### Cellular iron and calcium concentration measurement

Flame atomic absorption spectrometry (FAAS) is a technique for measuring the amount of chemical elements in samples. In all experiments, Caco-2 cells were seeded in 6-well plate at 3 × 10^4^ cells/well for the total of 15 days prior to FAAS. For iron treatment, Caco-2 cells were maintained in normal growth medium for 14 days before treated with 3 mM FAC in presence or absence of 0.5 mM ascorbic acid for 24 hours. Otherwise, Caco-2 cells were pre-treated with 500 nM hepcidin for 6 hours before exposing to 3 mM FAC together with 0.5 mM ascorbic acid for 24 hours, followed by FAAS. For hepcidin treatment, the basal level of cellular iron and calcium in Caco-2 cells were measured after being exposed to 500 nM hepcidin for 30 hours. For 1,25(OH)_2_D_3_ treatment, Caco-2 cells were maintained in normal growth medium until day 12; then, they were treated with 10 nM 1,25(OH)_2_D_3_ for 72 hours. For cells exposed to CaCl_2_, they were incubated with 12 mM CaCl_2_ in the last 24 hours prior to FAAS. In the cells treated with hepcidin together with 1,25(OH)_2_D_3_, Caco-2 cells were pre-treated with 10 nM 1,25(OH)_2_D_3_ alone for 48 hours; then, 500 nM hepcidin was applied in the last 30 hours before FAAS. 12 mM CaCl_2_ was applied in the last 24 hours before FAAS as mentioned previously. Cells were collected by cell scraper after washing twice with sterile phosphate buffer saline (PBS). Cell lysates were centrifuged at 6,500 rpm for 10 minutes then resuspended in 250 μL of ultrapure water for sonication. After that, the samples were digested with 65% nitric acid (HNO_3_) (Alchimica, UK) and 30% hydrogen peroxide (H_2_O_2_) (Merck) by Ethos UP MAXI-44 microwave digester (Milestone, CT, USA). Subsequently, sample volume was adjusted by ultrapure water before cellular iron or calcium measurement by FAAS (PinAAcle 900T Atomic Absorption Spectrometer, PerkinElmer, Waltham, MA, USA). Relative cellular iron or calcium values representing intestinal cell iron and calcium uptake capacity of intestinal cells were normalized to their total protein concentration and untreated control group.

### Quantitative Reverse Transcription Polymerase Chain Reaction (qRT-PCR)

Transcriptional mRNA expression was determined by qRT-PCR. Caco-2 cells were seeded at 4.2 × 10^5^ cells/well in 6-well plate (Corning, Glendale, AZ, USA). For hepcidin treatment, the cells were exposed to 500 nM hepcidin for 24 hours before cell pellet collection. On the other hand, 10 nM 1,25(OH)_2_D_3_ was introduced to the cells 6 hours after plating, and the cells were grown in 1,25(OH)_2_D_3_ containing media for 72 hours. Cell pellet was collected from all experimental groups at 72 hours after plating. Cell pellets were harvested by scraping. Total RNA from cell lysate was extracted by using Trizol reagent (Invitrogen, Carlsbad, CA, USA). RNA concentration was measured by using NanoDrop-2000c spectrophotometer (Thermo Fisher Scientific, Waltham, MA, USA). Next, RNA was converted to cDNA by using iScript reverse transcription supermix (Bio-rad, Hercules, CA, USA) with the thermal cycler (MJ Mini Thermal Cycler, Bio-rad). Quantitative reverse transcription polymerase chain reaction (qRT-PCR) was amplified by Bio-rad MiniOpticon using SsoFast EvaGreen Supermix (Bio-rad). PCR was operated with 3 steps including enzyme activation and DNA denaturation step at 95°C for 40 seconds, annealing step for 30 seconds and extension step at 65°C for 5 seconds. Primer sequences for each gene are indicated in Supplementary data (S1 Table). Samples were analyzed in at least three biological replicates. Relative gene expression was calculated from 2^–ΔCt^ value by using human GAPDH as a house-keeping gene, and 2^–ΔΔCt^ value as compared to the untreated controls.

### Western blot analysis

Protein level of certain proteins was evaluated by western blotting. Caco-2 cells were seeded at 4.2 × 10^5^ cells/well in 6-well plate and treated with either 500 nM hepcidin for 24 hours or 10 nM 1,25(OH)_2_D_3_ for 72 hours before collecting by scraping. Total protein was extracted by radioimmunoprecipitation assay (RIPA) buffer containing 50 mM Tris-HCl (Vivantis Technologies, Selangor Darul Ehsan, Malaysia), 1% TritonX-100 (Applichem, Darmstadt, Germany), 0.25% deoxycholate (Merck, Kenilworth, NJ, USA), 150 mM NaCl (Daejung chemicals, Gyeonggi-do, Korea) at pH 7.4 supplemented with 1× protease inhibitor (Roche, Mannheim, Germany) for 1 hour on ice. The concentration of protein samples was determined by using BCA protein assay kit (Thermo Fisher Scientific). Forty micrograms of proteins were separated by SDS-PAGE with 10% polyacrylamide gel at 100 volts for 120 minutes. After that, all proteins were transferred to nitrocellulose membrane (GE Healthcare Limited, Little Chalfont, Buckinghamshire, UK) at 12 volts for 120 minutes. Membrane was incubated with specific primary and secondary antibodies diluted in blocking solution at 4°C overnight and 2 hours at room temperature, respectively. Specific primary antibodies used in this study included mouse anti-divalent metal transporter1 (DMT1) monoclonal antibody (ab55735; Abcam, Cambridge, MA, USA), rabbit anti-solute carrier family 40 member 1 (SLC40A1; ferropotin, FPN) polyclonal antibody (ab58695; Abcam) and rabbit anti-β-actin monoclonal antibody (A2066; Sigma-Aldrich). Secondary antibodies used in this study included goat anti-rabbit (7074) and goat anti-mouse IgG (7076) conjugated with horseradish peroxidase (Cell signaling technology, Danvers, MA, USA). Proteins were monitored by Luminata crescendo western HRP substrate chemiluminescence (ECL; Millipore corporation, Billerica, MA, USA) exposed to Hyperfilm (GE Healthcare). Original blot images are presented in S1 Raw images, according to the journal’s guideline. Protein band intensities were quantified by using ImageJ software (National Institutes of Health). Relative protein level was calculated from band intensity of each protein normalized to its own actin and the control group, respectively.

### Statistical analysis

The results are expressed as means ± standard error of mean (SEM). The average values were calculated from at least three biological replicates from independent experiments. The probability values (*p*-values) between control and treatment groups in all experiments were analyzed by non-parametric test. The results were analyzed by one-tailed Mann-Whitney U test or Kruskal-Willis with Dunn’s Multiple Comparison Test. The critical significant level for all statistic tests was *p* < 0.05. All data were analyzed by GraphPad Prism 9 (GraphPad Software Inc., San Diago, CA, USA).

## Results

### Ascorbic acid-enhanced intestinal cell iron uptake was significantly reduced by hepcidin

**Fig 1:**
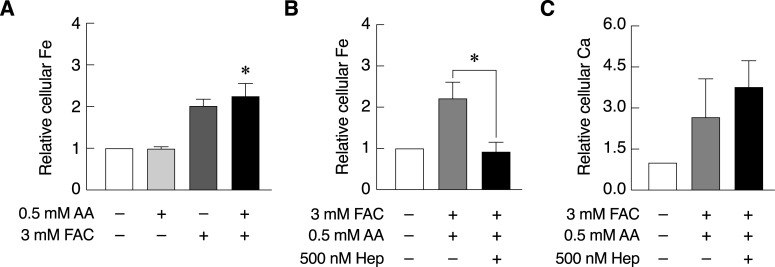
Ascorbic acid-enhanced intestinal cell iron uptake was significantly reduced by hepcidin. (*A*) Cellular iron in Caco-2 cells were measured by FAAS after exposed to 0.5 mM ascorbic acid, 3 mM FAC or their combination for 24 hours (n = 4), and (*B*) effects of hepcidin on cellular iron level under iron uptake stimulation was investigated in Caco-2 cells. The Caco-2 cells were pre-treated with 500 nM hepcidin for 6 hours and exposed to 3 mM FAC together with 0.5 mM ascorbic acid for 24 hours (n = 3). (*C*) Cellular calcium level from the same sample groups as shown in (B) were also measured by FAAS (n = 3). Data was analyzed from at least three independent biological experiments as indicated (n). *P*-value was calculated using the Kruskal-Willis with Dunn’s Multiple Comparison Test or one-tailed Mann-Whitney U Test. *P < 0.05 as compared to untreated sample.

Whether hepcidin-induced intestinal calcium absorption and its inhibitory effect on intestinal iron absorption could be replicated in other conditions beside thalassemia is not known. Therefore, this experiment aimed to investigate the effects of hepcidin on cellular iron and calcium uptake ability of enterocytes under iron transport stimulation. Ascorbic acid was used as an intestinal iron absorption enhancer, and ferric ammonium citrate (FAC) was used as a ferric donor in this experiment. The results showed that 3 mM FAC together with ascorbic acid treatment significantly increased cellular iron level as compared to control group indicating the higher cellular iron uptake capacity in Caco-2 cells (Fig 1A). Increased cellular iron level induced by FAC and ascorbic acid treatment was markedly abolished by hepcidin (Fig 1B). To determine the effects of hepcidin on intestinal cell calcium uptake under iron transport stimulation, cellular calcium level from the same sample groups with Fig 1B was also determined. Even though the increased trend of cellular calcium in Caco-2 cells treated with hepcidin was observed, our results showed that hepcidin did not significantly induce cellular calcium uptake in Caco-2 cells under iron transport stimulation (Fig 1C). Our results demonstrated that while hepcidin significantly suppressed intestinal cell iron uptake, it could not markedly elevate intestinal cell calcium uptake in Caco-2 cells under iron uptake stimulation.

### Hepcidin decreased basal cellular iron level but increased the basal cellular calcium level in Caco-2 cells

**Fig 2:**
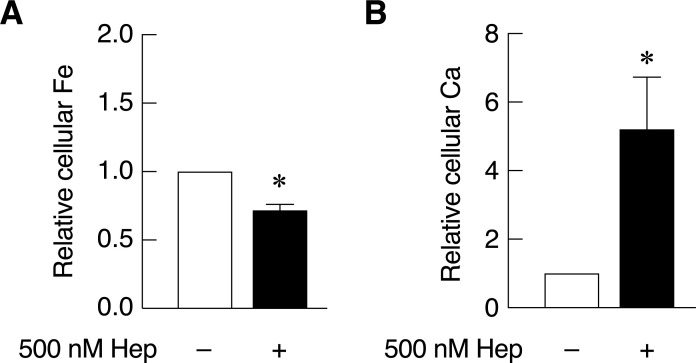
Hepcidin suppressed cellular iron uptake but activated cellular calcium uptake in Caco-2 cells under physiological condition. (*A*) The basal level of cellular iron and (*B*) cellular calcium in Caco-2 cells upon hepcidin treatment. The same sample groups were measured by FAAS after exposed to 500 nM hepcidin for 30 hours; data was analyzed from three independent biological experiments (n = 3). *P*-value was calculated using one tailed Mann-Whitney U Test. *P < 0.05 as compared to control group.

As shown in Fig 1B and 1C, hepcidin could suppress intestinal cell iron uptake but induce intestinal cell calcium uptake under iron uptake stimulation by ascorbic acid and FAC. However, the effects of hepcidin on the basal cellular iron and calcium level are not known. This experiment aimed to investigate the effects of hepcidin on the basal level of iron and calcium in enterocytes without extracellular iron or calcium treatment. The results showed that hepcidin significantly decreased cellular iron level in Caco-2 cells (Fig 2A). In contrast, hepcidin greatly enhanced cellular calcium in Caco-2 cells even in the absence of neither extracellular iron nor ascorbic acid (Fig 2B). Altogether, our results showed that hepcidin induced intestinal calcium cell uptake under physiological condition but not under iron transport stimulation condition.

### Hepcidin decreased iron transporter and iron transport-associated protein expression, but did not alter the expression of calcium transporter and calcium transport-associated proteins

**Fig 3:**
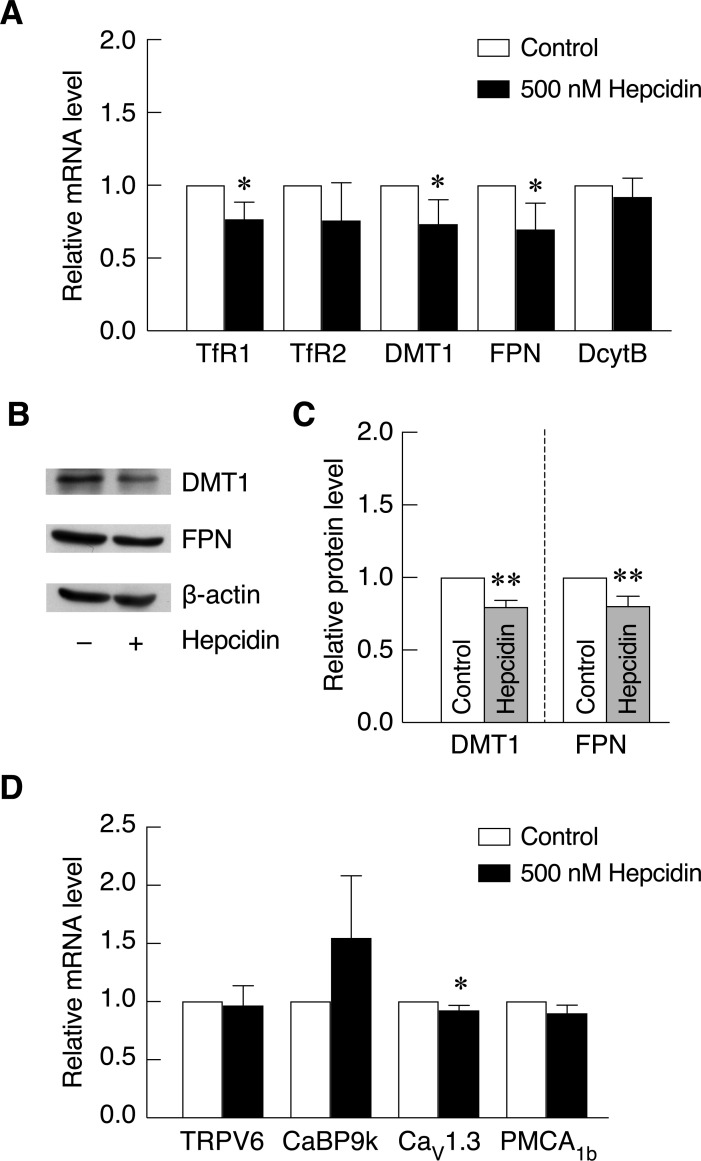
Hepcidin decreased DMT1 and FPN at mRNA and protein level but did not alter most calcium transporter and calcium binding protein expression at mRNA level. mRNA and protein expression of iron transporter and iron transport-associated proteins in Caco-2 cells after exposed to 500 nM hepcidin for 24 hours. (*A*) mRNA level was investigated by qRT-PCR and normalized by GAPDH and control groups (n = 3). (*B*) DMT1 and FPN protein expression was determined by western blot and normalized by β-actin, and (*C*) The quantitative data of protein band intensity was measured by Image J (n = 5). (*D*) The expression of calcium transporter and calcium binding protein was determined by qRT-PCR after exposed to 500 nM hepcidin for 24 hours (n = 3). Experiments were performed in three biological replicates each with three internal technical repeats for qRT-PCR experiments and five biological replicates for western blotting. *P*-value was calculated using one-tailed Mann-Whitney U Test. *P < 0.05, **P < 0.01 as compared to control group.

As a negative regulator for intestinal iron absorption, this study aimed to investigate and confirm the effects of hepcidin on the expression of iron transporter and iron transport-associated protein in Caco-2 cells. The results showed that hepcidin significantly decreased transferrin receptor (TfR1), DMT1 and FPN mRNA levels. While hepcidin did not significantly alter TfR2 and duodenal cytochrome B (DcytB) mRNA level, the slight reduction was also observed (Fig 3A). At translational level, DMT1 and FPN proteins in hepcidin treated Caco-2 cells were significantly decreased as compared to control cells (Fig 3B and 3C). With the positive effects of hepcidin on intestinal calcium transport and cellular uptake reported in previous and current studies ([15] and Fig 2B), effects of hepcidin on the expression of calcium transporter and calcium transport-associated protein was not known. In this study, the results showed that hepcidin did not significantly affect the expression of calcium transporter and calcium transport-associated protein including transient receptor potential vanilloid channel member 6 (TRPV6) and plasma membrane calcium ATPase (PMCA_1b_). Even though the results were not statistically different, the expression of calcium binging protein 9 (CaBP9k) was noticeably increased upon hepcidin treatment. On the other hand, there was a significant decrease in the expression of voltage-dependent calcium channel 1.3 (Ca_v_1.3), which is a non-canonical apical calcium channel (Fig 3D). These results showed that hepcidin significantly reduced the expression of iron transporters but had no significant effect on the canonical calcium transporter or calcium transport-associated protein.

### 1,25(OH)_2_D_3_ significantly increased cellular calcium uptake but had no effect on iron uptake in enterocytes

**Fig 4:**
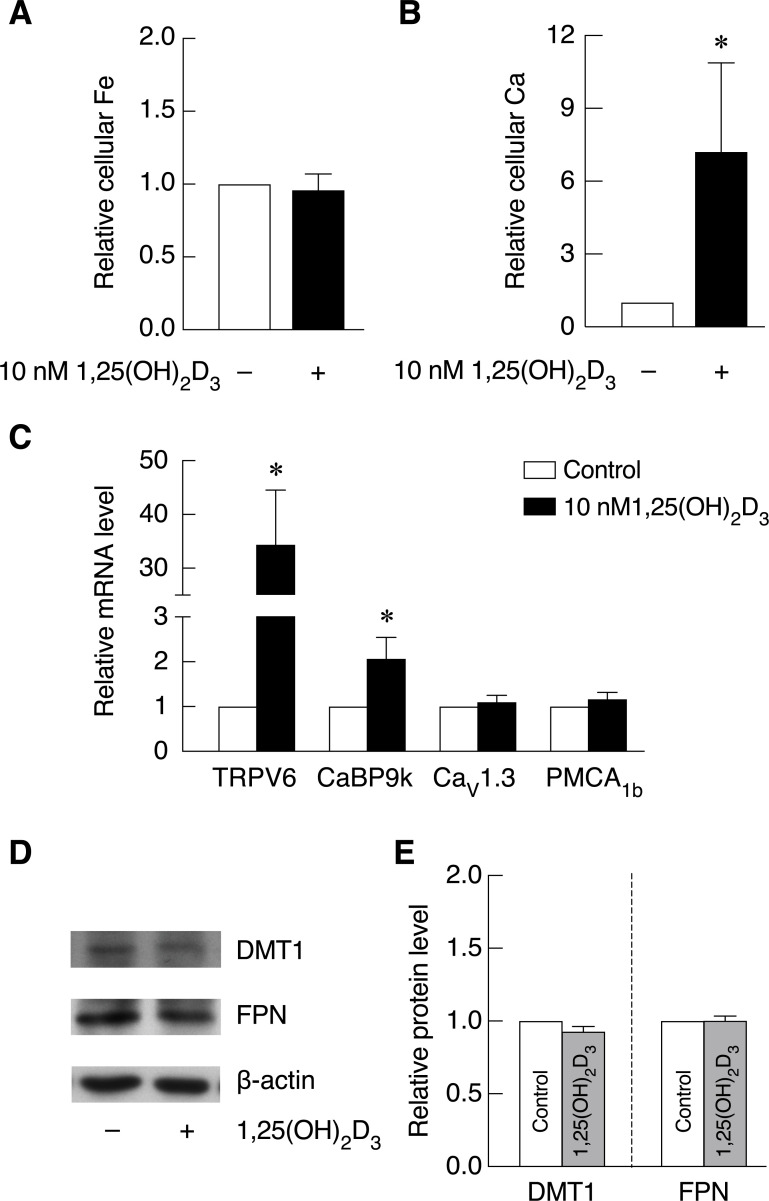
1,25(OH)_2_D_3_ strongly increased cellular calcium uptake and the expression of calcium transporter and calcium binding protein; however, it had no effect on cellular iron uptake nor iron transporter level in Caco-2 cells. (*A*) Cellular iron and (*B*) cellular calcium in Caco-2 cells of same sample groups were measured by FAAS after being treated with 1,25(OH)_2_D_3_ for 72 hours (n = 3). (*C*) mRNA expression of calcium transporter as well as calcium binding protein in Caco-2 cells was investigated after being exposed to 10 nM 1,25(OH)_2_D_3_ for 72 hours (n = 4). (*D*) DMT1 and FPN protein expression was determined by western blot (*E*) The quantitative data of protein band intensity as normalized by β-actin and control groups were analyzed by Image J (n = 3). Data was analyzed from at least three independent biological experiments as indicated (n). *P*-value was calculated using one-tailed Mann-Whitney U Test. *P < 0.05 as compared to control group.

As a positive regulator of calcium absorption, whether 1,25(OH)_2_D_3_ also affect intestinal cell iron uptake is not known. This study aimed to elucidate the effects of 1,25(OH)_2_D_3_ on cellular calcium and iron uptake ability in Caco-2 cells. Our results showed that 1,25(OH)_2_D_3_ markedly increased cellular calcium level in Caco-2 cells (Fig 4B). When cellular iron in the same groups of samples was determined, our results showed that 1,25(OH)_2_D_3_ did not alter cellular iron level in Caco-2 cells (Fig 4A). Taken together, our results showed that 1,25(OH)_2_D_3_ increased cellular calcium level but did not alter cellular iron level in Caco-2 cells indicating that 1,25(OH)_2_D_3_ induced intestinal cell calcium uptake but had no effect on intestinal cell iron uptake.

### 1,25(OH)_2_D_3_ induced the expression of calcium transporter and calcium transport-associated protein but did not alter DMT1 and FPN protein level

In this experiment, effects of 1,25(OH)_2_D_3_ on the expression of calcium transporter and calcium transport-associated protein were studied to verify the positive effects of 1,25(OH)_2_D_3_ on intestinal calcium transport in Caco-2 cells. As shown in Fig 4C, 1,25(OH)_2_D_3_ significantly increased mRNA expression of TRPV6 and CaBP9k. In addition, an increase in Ca_v_1.3 and PMCA_1b_ expression was found in 1,25(OH)_2_D_3_-treated groups (Fig 4C). We further showed that either DMT1 or FPN protein expression was not affected by 1,25(OH)_2_D_3_ treatment (Fig 4D and 4E). These results showed that 1,25(OH)_2_D_3_ has positive effects on calcium transporter gene expression, but had no effect on DMT1 and FPN protein level in Caco-2 cells.

### Hepcidin could not rescue intestinal cell calcium uptake, which was diminished by high CaCl_2_ concentration

**Fig 5:**
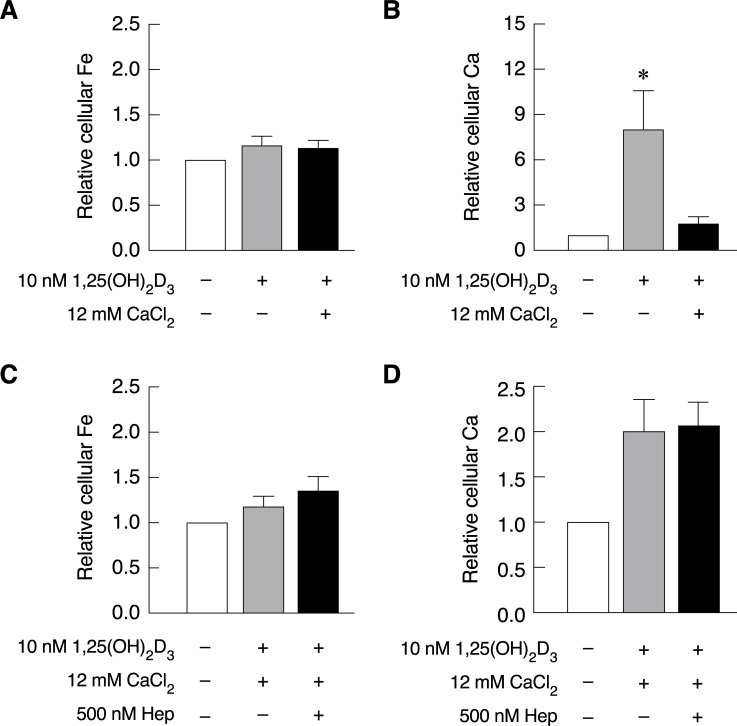
1,25(OH)_2_D_3_-induced intestinal cell calcium uptake was greatly abolished by high extracellular CaCl_2_ concentration, and this phenomenon could not be recovered by hepcidin. (*A*) Cellular iron and (*B*) cellular calcium in Caco-2 cells of same sample groups were measured by FAAS after exposed to 10 nM 1,25(OH)_2_D_3_ for 72 hours in the presence or absence of 12 mM CaCl_2_ for 24 hours. (*C*) Cellular iron uptake and (*D*) calcium uptake ability in Caco-2 cells of same samples were measured by FAAS under calcium uptake suppression by 12 mM CaCl_2_ in the presence or absence 500 nM hepcidin for 30 hours; data was analyzed from three independent biological experiments (n = 3). *P*-value was calculated using the Kruskal-Willis with Dunn’s Multiple Comparison Test. *P < 0.05 as compared to untreated samples.

Our previous experiment showed the effects of hepcidin and 1,25(OH)_2_D_3_ treatment on intestinal cell iron and calcium uptake under physiological and iron uptake stimulated conditions (Figs 1, 2 and 4). However, the role of hepcidin in intestinal cell calcium uptake under other calcium uptake suppression condition is still unknown. In iron uptake experiment, 1,25(OH)_2_D_3_ in the presence or absence of 12 mM CaCl_2_ did not change cellular iron level in Caco-2 cells (Fig 5A). Cellular calcium levels representing calcium uptake activity in Caco-2 cells treated with 1,25(OH)_2_D_3_ with or without CaCl_2_ are presented in Fig 5B. The results showed that 1,25(OH)_2_D_3_ treatment alone significantly increased cellular calcium in Caco-2 cells. However, this phenomenon was completely abolished by high CaCl_2_ concentration (Fig 5B), which is a normal feedback response to prevent excessive calcium hyperabsorption [31, 32]. Our results indicated that high extracellular calcium concentration suppressed intestinal cell calcium uptake, but had no effect on iron uptake. Results in Figs 1C and 2B showed that hepcidin treatment significantly increased cellular calcium level under physiological conditions, but not under a stimulated iron uptake condition. This experiment also aimed to examine whether the positive effects of hepcidin on intestinal cell calcium uptake could recover the CaCl_2_-inhibited intestinal cell calcium uptake. However, our results showed that cellular calcium could not be rescued by hepcidin from calcium uptake suppression by 12 mM CaCl_2_ (Fig 5D). Cellular iron level representing iron uptake activity in the same groups of samples in Fig 5D was also determined. The results showed that both conditions did not alter cellular iron level in Caco-2 cells (Fig 5C). Taken together, our results demonstrated that high concentration of CaCl_2_ suppressed 1,25(OH)_2_D_3_-induced cellular calcium uptake in Caco-2 cells, and hepcidin could not rescue intestinal cell calcium uptake in this scenario.

## Discussion

Iron overload-associated osteopenia and bone fracture are found in several diseases, such as thalassemia, which is an inherited anemia disease caused by underproduction of globin proteins [5–7, 33]. Previous study suggested that extracellular iron inhibited osteoblast cell survival and induced osteoblast cell death [2, 4, 34, 35]. In addition to the direct deleterious effects of iron on bone cells and bone remodeling process, intestinal calcium malabsorption has also been reported in thalassemia [1, 8, 9, 11]. Interestingly, Kraidith et al. have reported that calcium malabsorption could be improved by the intestinal iron transport suppressor, hepcidin [15]. Nevertheless, thalassemia is an inherited disease with several complications including anemia, iron overload, aberrant liver function and abnormal vitamin D metabolism. All of which could affect intestinal iron and calcium absorption [1, 25, 26]. Thereby, it is in question whether the positive effect of hepcidin on intestinal calcium uptake found in thalassemia also occurs in other conditions. This study aimed to elaborate if the same phenomenon could be seen in other conditions including physiological, iron uptake stimulation and calcium uptake suppression.

While normal serum iron levels have been reported to be 10–30 μM, the concentration over 50 μM was detected in iron overload patients [36, 37]. Therefore, FAC in a range of 100–200 μM was used to represent iron overload condition in previous studies [2, 4]. The normal extracellular calcium levels are within the range of 2.1–2.6 mM [38]. However, the higher concentrations of FAC and CaCl_2_ was used in this study to mimic dietary iron and calcium in the lumen of gastrointestinal tract, which was exposed to much higher concentrations than serum. Similar study also showed that 1.5 mM of FeCl_3_ significantly increased intracellular iron in Caco-2 cells as measured by electrothermal atomic absorption spectrometer [39]. Therefore, 3 mM FAC was used in the present study, and found to induce cellular iron uptake in Caco-2 cells in the presence of ascorbic acid (Fig 1A). In addition, our previous study has showed that high concentration of CaCl_2_ inhibited 1,25(OH)_2_D_3_-induced calcium transport in Caco-2 cells [40]. Thus, 12 mM CaCl_2_ was herein used to examine the effects of hepcidin under intestinal cell calcium uptake suppression by high concentration of CaCl_2_. Physiological serum level of hepcidin was reported to be 2.3 nM, 7.8 nM and 6.5 nM for infants, male adults and female adults, respectively [41, 42], and the levels could be over 300 nM in critically ill patients with anemia and hemodialysis patients [43, 44]. Moreover, previous study showed that hepcidin at 200, 500 and 1000 nM was shown to significantly suppress transepithelial iron transport in Caco-2 cells; while, 50 nM hepcidin did not [22]. Therefore, 500 nM hepcidin was used in this study, and it significantly suppressed cellular iron uptake in Caco-2 cells in both physiological and stimulated iron uptake conditions (Figs 1B and 2A).

It has been reported that ascorbic acid increased intestinal cell iron uptake from gut lumen into enterocytes by many processes [45–48]. Our results confirmed the positive roles of ascorbic acid and negative effects of hepcidin on intestinal cell iron uptake (Fig 1). Similarly, we showed that ascorbic acid together with 3 mM FAC was the most effective condition to enhance cellular iron uptake in Caco-2 cells as compared to control, so this condition was used to study the effects of hepcidin on intestinal cell iron uptake under iron uptake stimulation. As shown in Fig 1B, ascorbic acid-induced intestinal cell iron uptake was completely abolished by hepcidin. Our results corresponded to other studies in rat brain and Caco-2 cells, where hepcidin suppressed cellular iron uptake and transport [18, 22, 49, 50]. In addition to its inhibitory role in intestinal cell iron uptake, hepcidin was also shown to regulate iron recycling in macrophages [19, 51, 52]. Interestingly, we found that ascorbic acid and FAC together can also slightly stimulate intestinal cell calcium uptake in Caco-2 cells, and hepcidin did not significantly induce cellular calcium uptake (Fig 1C). Similar phenomenon has been reported in osteoblast cells (hFOB 1.19) under high environmental iron concentration where 200 μM FAC and hepcidin treatment significantly increased cellular calcium uptake in these cells, as determined by fluorescent labeling with Fluo-3/AM followed by flow cytometry [53]. Even though the underlying mechanism for this phenomenon is yet to be discovered, ROS-mediated mechanism was proposed. Moreover, a study in Caco-2 cells by using fluorescent labeling technique with Fluo-3/AM followed by fluorescent signal measurement under confocal laser scanning system also showed that low concentration of FAC (10 μM) significantly increased intracellular calcium in Caco-2 cells as compared to untreated control. However, this effect was suppressed to the similar level as untreated control when higher FAC concentration of 100 μM was used [54]. According to the previous study, we hypothesized that 3 mM FAC alone should not induce intestinal cell calcium uptake in Caco-2 cells in the present study.

To verify whether the same phenomenon could be seen in normal physiological conditions, the effects of hepcidin on intestinal cell calcium and iron uptake were herein investigated in Caco-2 cells. We found that hepcidin significantly decreased cellular iron uptake, but strikingly increased cellular calcium uptake ability, indicating its potential to modulate both intestinal iron and calcium transport systems in intestinal cells (Fig 2). These findings have suggested the possible application of hepcidin to concurrently help alleviate the aberrant iron hyperabsorption and ineffective calcium absorption. While the mechanism behind hepcidin-induced intestinal cell calcium uptake is not known, some studies also revealed similar phenomena in other types of cells. For example, in osteoblasts, hepcidin increased cellular calcium level through L-type calcium channel, which also expressed and played an important role in intestinal calcium transport [55]. In addition, study in hFoB1.19 osteoblast-like cells also showed that positive effects of hepcidin on cellular calcium level was associated with the release of stored calcium from endoplasmic reticulum [53]. Hence, these results suggested that the increased cellular calcium in hepcidin-treated osteoblasts could come from both calcium uptake through calcium channel and calcium release from ER into the cytoplasmic compartment. Regarding the present study, since FAAS was used to quantify the total level of cellular calcium concentration, the increased cellular calcium level in hepcidin-treated Caco-2 cells could represent augmented intestinal cell calcium uptake ability not the change in subcellular localization of calcium in the cytoplasm. Therefore, results from our study demonstrated the ability of hepcidin to enhance cellular calcium uptake in intestinal cells. Regardless, this phenomenon could also rely on both the effect of hepcidin on calcium transporter directly and on the release of stored calcium from endoplasmic reticulum. In addition to the alteration of subcellular localization of calcium, the depletion of calcium stored in ER could trigger the activity of plasma membrane calcium transporter, leading to the enhanced calcium uptake and increasing cellular calcium level through the process called Ca^2+^ release-activated Ca^2+^ channel (CRAC). Nevertheless, whether the hepcidin-induced intestinal cell calcium uptake require extracellular iron is not known. A previous study showed that, in hepcidin-exposed osteoblasts, an increase in intracellular calcium level was further enhanced under high extracellular iron condition [53]. We, therefore, postulated that the presence of extracellular iron could be beneficial for hepcidin-induced cellular calcium uptake in our model. However, more studies are needed to verify these hypotheses.

The suppression of intestinal cell iron uptake by hepcidin could be a result of iron transporter and iron transport-associated protein downregulation. Furthermore, the effects of hepcidin on the alteration of calcium transporters and calcium-associated protein have never been examined. To elaborate these questions, the expression of iron and calcium transporters and some associated genes in Caco-2 cells upon hepcidin treatment was examined. Our results showed that hepcidin significantly downregulated several iron transporters and iron transport-associated proteins at mRNA and protein level, especially TfR1, DMT1 and FPN (Fig 3). Corresponding to these results, previous studies also showed that hepcidin suppressed DMT1 and FPN expression in Caco-2, brain microvascular endothelial cells (BMECs) and BKO mice [15, 18, 22, 49, 50, 56]. It has been shown that hepcidin could directly interact with FPN on the basolateral side of enterocytes to activate FPN internalization and degradation [57–59]. Nevertheless, the mechanism underlying hepcidin-induced DMT1 downregulation is still unclear because DMT1 is predominantly expressed on the apical side of intestinal cells.

Since the positive effects of hepcidin on intestinal cell calcium uptake under iron exposure was observed in this study, its effect on DMT1 and FPN expression was not investigated. Even though our previous results in thalassemic mice showed that hepcidin only downregulated DMT1 but not FPN in these mice [15], another study from non-thalassemic mice showed that hepatic hepcidin knockdown was shown to correlate with the increased level of duodenal DMT1 and FPN both in transcriptional and translational levels [60]. Similarly, other studies in rats also showed the inverse correlation between hepatic hepcidin mRNA level and duodenal FPN mRNA level under lipopolysaccharides (LPS) treatment and high iron diet [61, 62]. Therefore, we hypothesized that the different response seen in hepcidin-treated thalassemia mice may result from the different FPN regulation in thalassemia, which remains to be deciphered. According to these studies, we hypothesized that hepcidin effects under iron overload could be similar to its effects under physiological conditions. Since dietary iron is composed of heme iron and nonheme iron, the intestinal iron absorption occurs through heme carrier protein 1 (HCP1) and DMT1 [63]. While TfR1 functions in transferrin-bound iron uptake in several types of cells including macrophages, it has been shown to have a unique role in intestinal cell proliferation and maintaining intestinal homeostasis without affecting intestinal iron absorption [64]. Therefore, we postulate that the downregulation of intestinal TfR1 under hepcidin treatment was not associated with ion absorption alteration seen in this experiment.

When effects of hepcidin on the expression of calcium transporters and calcium transport-associated protein was investigated, our results showed that hepcidin did not cause a significant change in calcium transporter expression with an exception for a reduction in Ca_v_1.3 (Fig 3). Even though the results did not reach statistical significance, noticeable increase in CaBP9k expression was seen in hepcidin treated cells. From our results, hepcidin-induced intestinal cell calcium uptake was not likely to come from the increased expression of calcium transporters or its associated proteins. Nonetheless, changes in the protein expression levels cannot be ruled out, and functionality of these transporters remains to be investigated. As a well-known potent activator for intestinal calcium transport [65–68], it is worth mentioning that standard 1,25(OH)_2_D_3_ treatment failed to recover intestinal calcium transport in thalassemic mice. However, hepcidin could effectively alleviate intestinal calcium absorption [15]. Previous study showed that 25(OH)_2_D_3_ suppressed hepcidin expression at mRNA level in hepatocyte and monocytes. This led to the increased FPN expression in these cells. Similar phenomenon was also observed as vitamin D supplementation resulted in increased serum level of hepcidin in healthy individuals [69]. To examine the effects of 1,25(OH)_2_D_3_ on iron transport system in intestinal cells, both intestinal cell iron uptake and iron transporter expression were examined upon 1,25(OH)_2_D_3_ treatment in this study. Our results showed that 1,25(OH)_2_D_3_ could stimulate intestinal cell calcium uptake without affecting cellular iron uptake in Caco-2 cells (Fig 4). Therefore, increased intestinal cell calcium uptake by 1,25(OH)_2_D_3_ did not affect intestinal cell iron uptake and could not recover iron hyperabsorption as seen from hepcidin. In addition, our results and previous studies confirmed that 1,25(OH)_2_D_3_ greatly induced the expression of calcium transporters and calcium transport-associated protein, especially TRPV6 (Fig 4 and [70–73]). However, 1,25(OH)_2_D_3_ did not alter DMT1 and FPN protein level in Caco-2 cells (Fig 4). These results corresponded to the unchanged cellular iron uptake capacity in Caco-2 cells after exposed to 1,25(OH)_2_D_3_ as shown in Figs 4 and 5. To further investigate whether hepcidin could also rescue intestinal cell calcium uptake in other calcium uptake suppression condition, effects of hepcidin on cellular iron and calcium in enterocytes were examined under 12 mM CaCl_2_, high concentration of extracellular calcium shown to inhibit 1,25(OH)_2_D_3_-induced calcium transport [40]. The results showed that 12 mM CaCl_2_ markedly suppressed 1,25(OH)_2_D_3_-induced intestinal cell calcium uptake in Caco-2 cells (Fig 5). The inhibitory effects of high concentration of CaCl_2_ on intestinal calcium transport was also reported in previous study from our group suggesting that the exposure of high calcium could inhibit transcellular intestinal calcium transport, potentially through the upregulation of FGF-23 [40, 74]. However, the inhibitory effect of CaCl_2_ on intestinal cell calcium uptake could not be rescued by hepcidin treatment (Fig 5). Similar to Fig 4, 1,25(OH)_2_D_3_ treatment in the presence or absence of CaCl_2_ and hepcidin did not affect intestinal cell iron uptake. These results suggested that hepcidin could not be used to alleviate intestinal calcium uptake suppression under high-calcium condition. Hence, the molecular mechanism for hepcidin-induced intestinal calcium absorption is crucial to determine specific conditions where hepcidin could be used.

In conclusions, our study showed that hepcidin could induce intestinal cell calcium uptake; on the other hand, its negative effects on iron transport system were also confirmed. Hepcidin suppressed intestinal cell iron uptake by inhibiting iron transporter and iron transport-associated protein expression, especially in DMT1 and FPN. Hepcidin-enhanced intestinal cell calcium uptake could be seen in physiological but not under iron uptake stimulation conditions. Moreover, hepcidin could not rescue cellular calcium uptake suppression by high calcium concentration. While the mechanism behind hepcidin-induced cellular calcium uptake in Caco-2 cells is unknown, our results also suggested that hepcidin-induced intestinal cell calcium uptake was not likely to result from the changes in calcium transporter expression. Moreover, the known calcium transport enhancer, 1,25(OH)_2_D_3_, did not affect intestinal cell iron uptake or iron transporter expression in Caco-2 cells. Although more studies are needed to investigate the mechanism underlying hepcidin-induced intestinal calcium transport, our study has provided the crucial information for the potential use of hepcidin that could concurrently help alleviate both iron hyperabsorption and impaired calcium absorption.

## Supporting information

S1 Table The *Homo sapiens* primers used in the qRT-PCR experiments. (PDF)

S1 Raw images Original blot images of Fig 3B and 3C. (PDF)

S2 Raw images Original blot images of Fig 4D and 4E. (PDF)
