# Supplementary material for: Hepcidin induces intestinal calcium uptake while suppressing iron uptake in Caco-2 cells
Source: PLoS One. 2021 Oct 13;16(10):e0258433. doi: 10.1371/journal.pone.0258433 (PMC8513844; doi:10.1371/journal.pone.0258433)
Supplement: S1 Table — (PDF) [file pone.0258433.s001.pdf]

Table S1. The *Homo sapiens* primers used in the qRT-PCR experiments

Genes	Primer sequences (Forward/Reverse)	Annealing temperature (°C)
**Calcium transport related genes**		
Plasma membrane Ca^{2+} ATPase 1b ($PMCA_{1b}$)	5′-AGAAGGTGGAGATGGTGATGA-3′<br>5′-CCCAGAAGGTGTCAATGACA-3′	53
Calcium binding protein domain 9 (CaBP9k)	5′-TAGCTGTTTCACTATTGGGCA-3′<br>5′-TTCATCCTTTGACAACTGGTCT-3′	52
Transient receptor potential vanilloid channel member VI (TRPV6)	5′-TCTGACTGCGTGTTCTCAG-3′<br>5′-ACATTCCTTGGCGTTCAT-3′	51
Calcium Voltage-dependent channels ($Ca_v1.3$)	5′-TGATCCAAGTGGAGCAGTCA-3′<br>5′-GTGTGAAAGTCCGGTAGGAGA-3′	54
**Iron transport related genes**		
Transferrin receptor1 (TfR1)	5′-CAGGAACCGAGTCTCCAGTGA-3′<br>5′-CTTGATGGTGCCGGTGAAGT-3′	53
Transferrin receptor2 (TfR2)	5′-TCTCAGACCGTCTACCAGCG-3′<br>5′-CAGTAGGAAGGCCCCAGTGA-3′	52
Divalent Metal Transporter 1 (DMT1)	5′-AGCTGTCATCATGCCACACA-3′<br>5′-AGACTTCAACCACCTGCTCG-3′	52
Duodenal cytochrome B (DcytB)	5′-TGCATACAGTACATTCCCGCCAGA-3′<br>5′-ATGGAACCTCTTGCTCCCTGTTCA-3′	55
Ferroportin (FPN)	5′-CGTCATTGCTGCTAGAATCG-3′<br>5′-AGACTGAAATCAATACGAGC-3′	47
**Housekeeping gene**		
Glyceraldehyde 3-phosphate dehydrogenase (GAPDH)	5′-TTGTTGCCATCAATGACCC-3′<br>5′-ATTTTGGAGGGATCTCGCT-3′	49
