# Supplementary figures and images for: Hepcidin induces intestinal calcium uptake while suppressing iron uptake in Caco-2 cells
Source: PLoS One. 2021 Oct 13;16(10):e0258433. doi: 10.1371/journal.pone.0258433 (PMC8513844; doi:10.1371/journal.pone.0258433)

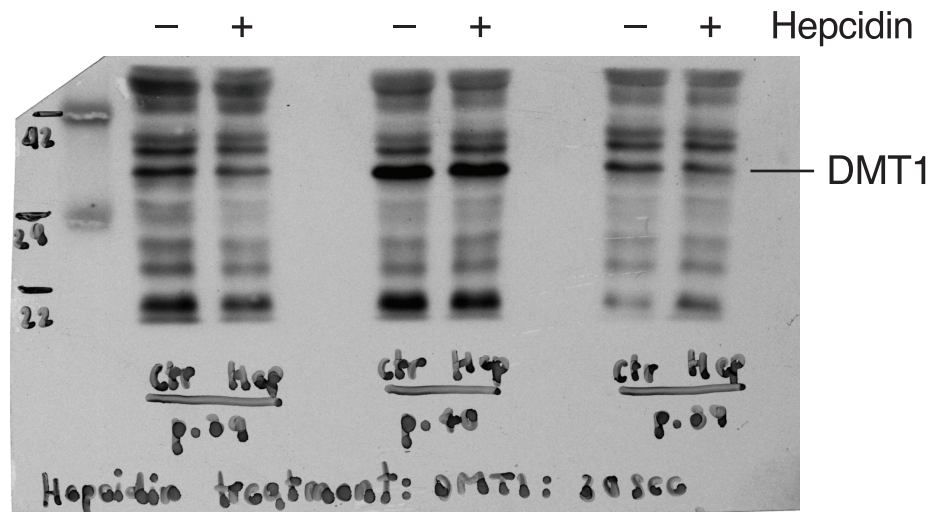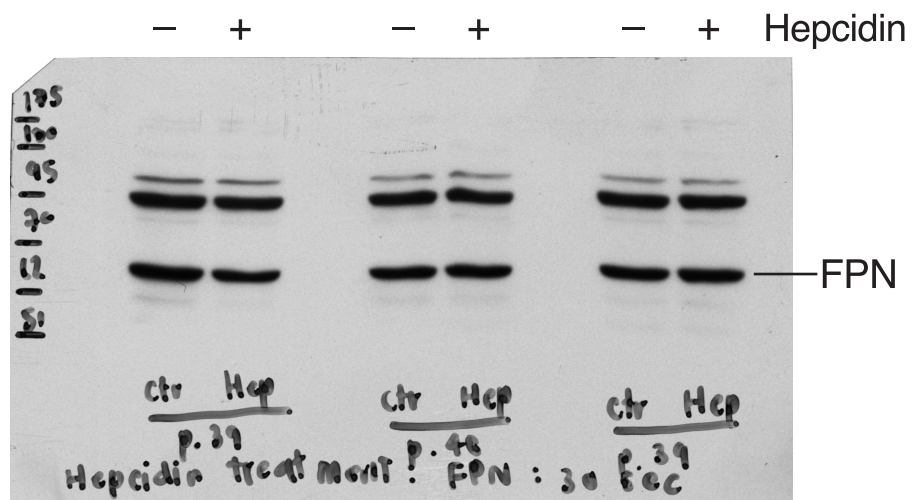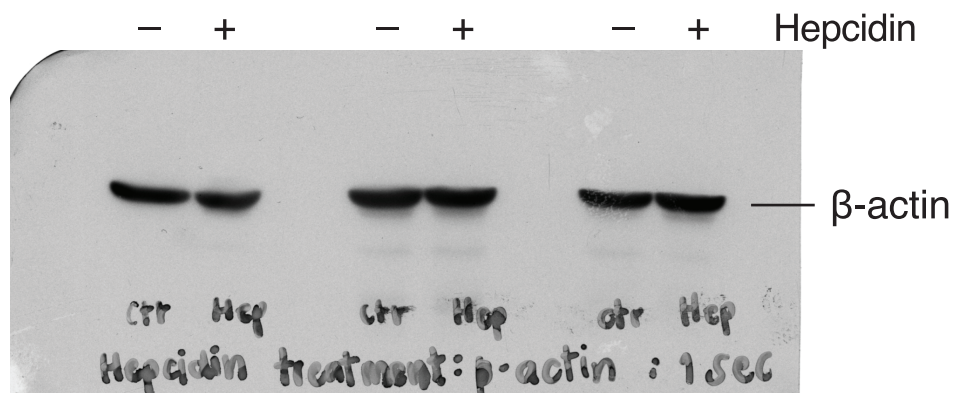

**Supplementary Figure S1 (Raw Images):** Phoaubon et al.  
Original blot images of Figs. 3B and 3C.

Supplement: S1 Raw images — (PDF) [file pone.0258433.s002.pdf]

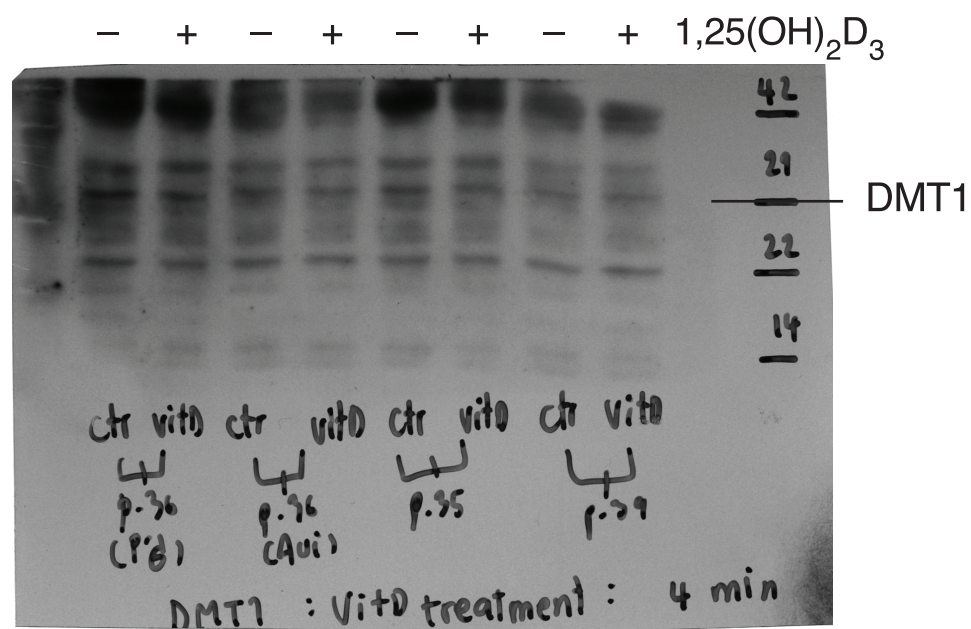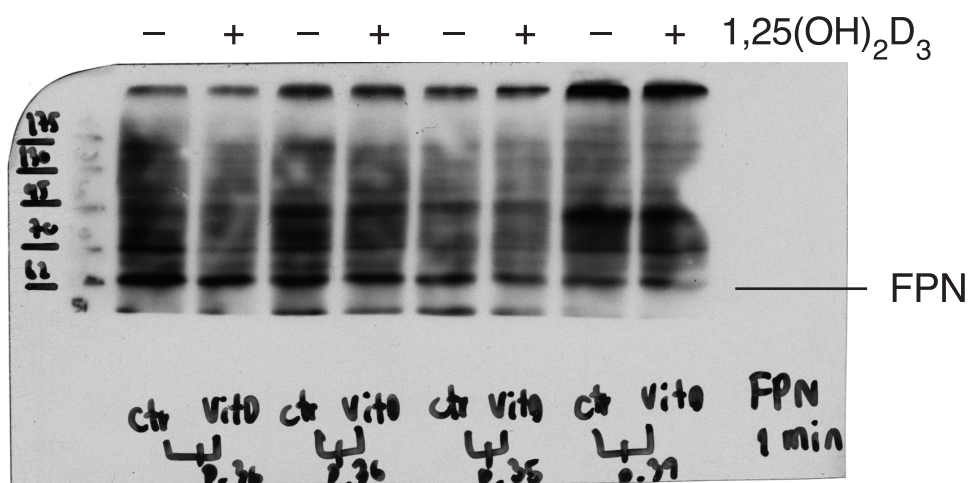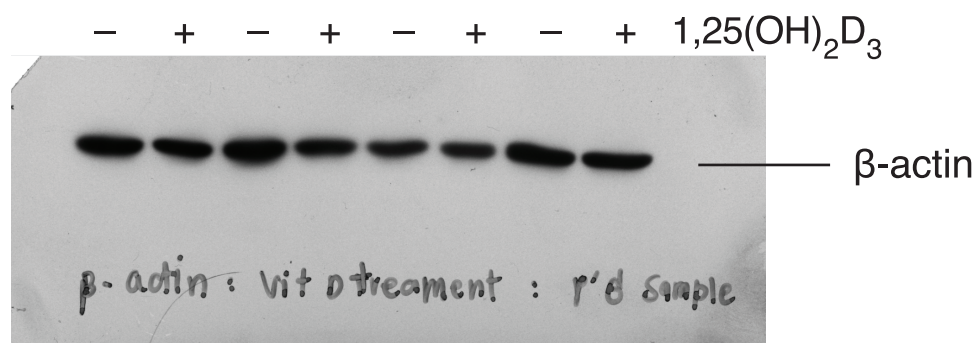

**Supplementary Figure S2 (Raw Images):** Phoaubon et al.  
Original blot images of Figs. 4D and 4E.

Supplement: S2 Raw images — (PDF) [file pone.0258433.s003.pdf]
